# Ramanujan’s Theta Functions and Parity of Parts and Cranks of Partitions

**DOI:** 10.1007/s00026-022-00615-1

**Published:** 2022-10-25

**Authors:** Koustav Banerjee, Manosij Ghosh Dastidar

**Affiliations:** 1 Research Institute for Symbolic Computation, Johannes Kepler University, Altenberger Strasse 69, 4040 Linz, Austria; 2 Technische Universität Wien, Wiedner Hauptstrasse 8–10/104, 1040 Wien, Austria

**Keywords:** Ramanujan’s theta functions, Partitions, Parity of parts, Cranks, 05A17, 11P83

## Abstract

In this paper, we explore intricate connections between Ramanujan’s theta functions and a class of partition functions defined by the nature of the parity of their parts. This consequently leads us to the parity analysis of the crank of a partition and its correlation with the number of partitions with odd number of parts, self-conjugate partitions, and also with Durfee squares and Frobenius symbols.

## Introduction

A *partition* of a positive integer *n* is a finite non-increasing sequence of positive integers $\pi _1,\pi _2,\dots ,\pi _r$ such that $\sum _{i=1}^{r}\pi _i=n$. The $\pi _i$ are called the parts of the partition. The partition $(\pi _1,\pi _2,\dots ,\pi _r)$ will be denoted by $\pi$, and we shall write $\pi \vdash n$ to denote that $\pi$ is a partition of *n*. The partition function *p*(*n*) is the number of partitions of *n*. A partition of *n* has a Durfee square of side *s* if *s* is the largest number such that the partition contains at least *s* parts with values $\ge s$. One of the more significant results in the elementary theory of partitions is the Euler’s fundamental and beautiful theorem:

### Theorem 1.1

[11, Theorem 1.1.10]. The number of partitions of a positive integer *n* into distinct parts equals the number of partitions of *n* into odd parts.

Consider the following refinement of Euler’s theorem which is stated above. Let *k* and *n* be positive integers with $k \ge 2$. Then the number of partitions of *n* into parts which are not multiples of *k*, denoted by *p*(*n*, *k*), equal to the number of partitions of *n* into parts with multiplicity of parts $<k$. For example, there are six partitions enumerated by *p*(5, 4) are $5,\ 3+2,\ 3+1+1,\ 2+2+1,\ 2+1+1+1,\ 1+1+1+1+1.$ For $k=2$, we retrieve Theorem 1.1.

Ramanujan [19, 20] investigated *p*(*n*), and discovered congruences in special arithmetic progressions such as:

1.1 $$\begin{aligned} p(5n+4)\equiv & {} 0 \ (\text {mod} \ 5),\nonumber \\ p(7n+5)\equiv & {} 0 \ (\text {mod} \ 7),\nonumber \\ p(11n+6)\equiv & {} 0 \ (\text {mod} \ 11). \end{aligned}$$

Define

$$\begin{aligned} \begin{aligned} (a;q)_{0}&:=1, \ \ \ (a;q)_{n}:=\prod _{k=0}^{n-1}(1-aq^k), \ n \ge 1;\\ (a;q)_{\infty }&:=\lim _{n\rightarrow \infty }(a;q)_{n}, \ \ |q|<1. \end{aligned} \end{aligned}$$

Ramanujan’s two-variable general theta function is defined as

1.2 $$\begin{aligned} f(a,b):= \sum _{n=-\infty }^{\infty }a^{n(n+1)/2}b^{n(n-1)/2}, \ \ |ab|<1. \end{aligned}$$

Three special cases of (1.2) are defined by, in Ramanujan’s notation

$$\begin{aligned} \begin{aligned} \phi (q)&:=f(q,q)=\sum _{n=-\infty }^{\infty }q^{n^2},\\ \psi (q)&:=f(q,q^3)=\sum _{n=0}^{\infty }q^{n(n+1)/2},\\ f(-q)&:=f(-q,-q^2)=\sum _{n=-\infty }^{\infty }(-1)^n q^{n(3n-1)/2}=(q;q)_{\infty }. \end{aligned} \end{aligned}$$

Besides the above three functions, Ramanujan defines a further one

$$\begin{aligned} \chi (q):=(-q;q^2)_{\infty }, \end{aligned}$$

which is not a theta function but it plays a prominent role in the theory of theta functions. Following Ramanujan’s definition (1.2), Jacobi’s famous triple product identity [1, Theorem 2.8]

$$\begin{aligned} \sum _{n=-\infty }^{\infty }q^{n^2}z^n=(-qz;q^2)_{\infty }(-q/z;q^2)_{\infty }(q^2;q^2)_{\infty }, \ \ |q|<1 \ \text {and} \ z\ne 0 \end{aligned}$$

takes the shape

1.3 $$\begin{aligned} f(a,b)=(-a,ab)_{\infty } (-b,ab)_{\infty } (ab,ab)_{\infty }. \end{aligned}$$

From [10, Entry 31, Equation (31.1)], it follows that we can express *f*(*a*, *b*) as the *n*-linear combination of theta functions in the following form

1.4 $$\begin{aligned} f(a,b)&=\sum _{r=0}^{n-1}a^{r(r+1)/2}b^{r(r-1)/2}f\nonumber \\&\quad (a^{n(n+1)/2+nr}b^{n(n-1)/2+nr},a^{n(n-1)/2-nr}b^{n(n+1)/2-nr}). \end{aligned}$$

For a more comprehensive analysis on Ramanujan’s theta function, we refer to [10, Chapter 16]. We shall subsequently present two results, namely Lemma 1.2 (resp. Lemma 1.3) for 5-dissection of $f(-q)$ (resp. $1/f(-q)$). Ramanujan defined what was later called the Rogers–Ramanujan continued fraction

1.5 $$\begin{aligned} R(q):={\frac{q^{1/5}}{1}}_{ \ +} \ {\frac{q}{1}}_{ \ +} \ {\frac{q^2}{1}}_{ \ +} \ {\frac{q^3}{1}}_{ \ +\dots } = q^{1/5} \frac{f(-q,-q^4)}{f(-q^2,-q^3)}, \ |q|<1. \end{aligned}$$

### Lemma 1.2

[11, p. 161 and p. 164]. If $$T(q):=\frac{q^{1/5}}{R(q)}=\frac{f(-q^2,-q^3)}{f(-q,-q^4)}$$,

1.6 $$\begin{aligned} T(q^5)-q-\frac{q^2}{T(q^5)}=\frac{(q;q)_{\infty }}{(q^{25};q^{25})_{\infty }}. \end{aligned}$$

### Lemma 1.3

[11, p. 165, Equation (7.4.14)].

1.7 $$\begin{aligned} \begin{aligned} \frac{1}{(q;q)_{\infty }}&= \frac{(q^{25};q^{25})^{5}_{\infty }}{(q^5;q^5)^{6}_{\infty }}\Biggl (T^{4}(q^5)+q T^{3}(q^5)+2q^2 T^{2}(q^5)+3q^3T(q^5)\\&\quad +5q^4-\frac{3q^5}{T(q^5)} +\frac{2q^6}{T^{2}(q^5)}-\frac{q^7}{T^{3}(q^5)}+\frac{q^8}{T^{4}(q^5)}\Biggr ).\\ \end{aligned} \end{aligned}$$

In 1944, Dyson [14] discovered a beautiful combinatorial interpretation for the congruences of *p*(*n*) modulo 5 and 7 by introducing the concept of the rank of integer partitions and later, Andrews and Garvan [7] defined and established the crank, hypothesized by Dyson, to give a combinatorial proof of congruence for *p*(*n*) modulo 11 (1.1).

### Definition 1.4

([7]). For a partition $\pi$, let $l(\pi )$ denote the largest part of $\pi$, $w(\pi )$ denote the number of 1s in $\pi$ and $\mu (\pi )$ denote the number of parts of $\pi$ that are larger than $w(\pi )$. The crank $c(\pi )$ is given by

$$\begin{aligned} c(\pi )= {\left\{ \begin{array}{ll} l(\pi ), &{}\quad \text {if}\, w(\pi )=0, \\ \mu (\pi )-w(\pi ), &{}\quad \text {if}\,w(\pi )>0.\\ \end{array}\right. } \end{aligned}$$

Let $c_{e}(n)$ (resp. $c_{o}(n)$) be the number of partitions of *n* with even (resp. odd) crank and further, let $c_{e,o}(n)$ be the difference between $c_{e}(n)$ and $c_{o}(n)$ [2, Equation (6.2)]. The study on $c_{e,o}(n)$ began with the work of Andrews and Lewis [8]. Further investigation on $c_{e,o}(n)$ which describes both combinatorial results and analytic ones which include Ramanujan type congruences modulo powers of 5 and classical asymptotic formula were introduced in the work of Choi, Kang, and Lovejoy [13]. We find in Andrews’ [2, Section 6] how Ramanujan’s third-order mock theta functions $\phi _{3}(q)$ and $\psi _{3}(q)$ also come into prominence in the study of classical ranks and cranks in partitions.

### Definition 1.5

([16]). The Frobenius symbol is obtained through extraction from the Ferrers graph of a partition $\pi$ as follows: We delete the diagonal of the Ferrers graph. If the diagonal is of length *j*, we form the top row of the Frobenius symbol using the nodes to the right of the diagonal and similarly form the bottom row from the nodes below the diagonal. The Frobenius symbol of $\pi$ is denoted by $\mathfrak {F}(\pi )$.

For instance, in the partition $\pi = (7,4,4,2,1)\vdash 18$, the Ferrers graph is 
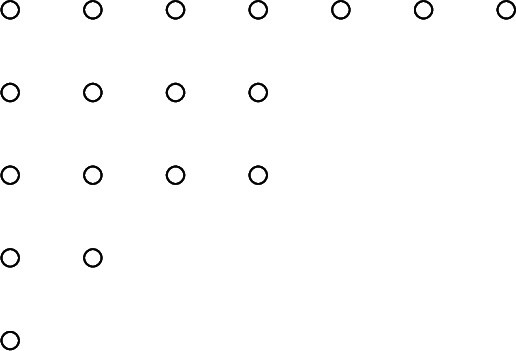
 and correspondingly the Frobenius symbol $\mathfrak {F}(\pi )$ is $$\begin{pmatrix} 6 &{}\quad 2 &{}\quad 1 \\ 4 &{}\quad 2 &{}\quad 0 \end{pmatrix}$$.

Ramanujan’s theta functions are the central theme of this paper. At the very outset, we establish a few partition identities where restrictions are imposed on the partition functions based on the parity of parts and their correlation to the aforementioned theta functions. A slew of investigations have been carried out on the parity study of partitions in recent years. Andrews’ [3] studied on the parts of partitions that are separated by parity, either all odd parts are smaller than all even parts or vice versa. Bringmann and Jennings-Shaffer [12] have extended the work of Andrews’ with a thorough *q*-series analysis that finally connects the parity study of partitions to the regime of partial theta functions, Ramanujan’s third-order mock theta function $\nu (q)$, and combinatorial interpretation by hook-type statistics in [9]. We will see how the parity biases of parts in partitions entangled with partitions with multiplicity of parts less than or equals to 4, denoted by *p*(*n*, 4), finally connect very naturally to Ramanujan’s theta functions. We undertake a detailed study on the parity of cranks through the lens of Ramanujan’s theta functions (see Theorems 1.6 and 1.7 below). Next, we examine the parity and associated congruence properties of the function delineating the difference between even and odd cranks of partitions (see Theorem 1.8). We prove a congruence modulo 5 for $c_{e,o}(n)$ by analyzing 5-dissection of Ramanujan’s theta functions which in turn shows that an arithmetic progression of the sequence $c_{o}(n)$ is divisible 10 (see Theorem 1.9), without using the machinations of modular forms, as given in [13, Theorem 1.2]. The novelty of Theorem 1.10 is that it identifies the odd crank enumeration of partitions with those partitions into odd number of parts and self-conjugate partitions through Liouville’s function $\lambda$. Following the work done in [6, 18], Theorem 1.11 springs up rather organically. Here, we count Frobenius symbols with restrictions on the entries and equate them to the enumeration of number of partitions with no parts that equal the size of the Durfee square of that partition, two ideas in the theory of partitions that are very rarely correlated.

The rest of this paper is organized as follows: in the remaining part of this section, we shall state all the main results, see Theorems 1.6–1.11. Before presenting the theorems, we shall provide all the necessary definitions, sometimes with examples, so as to ease the stating of the theorems. The proofs of Theorems 1.6–1.11 are given in Sect. 2.

We consider partitions whose odd parts unrestricted (resp. even parts distinct) tagged by couplet “*ou*” (resp. “*ed*”). Let $p_{ou,ed}(n)$ denote the number of partitions of *n* such that odd parts are unrestricted and even parts are distinct and $\mathcal {E}_{u}(n)$ denote the number of partitions of *n* such that even parts are unrestricted and each positive even integer smaller than the largest even part must appear as a part of the partition. As an instance, the six partitions enumerated by $p_{ou,ed}(5)$ are $5,\ 4+1,\ 3+2,\ 3+1+1,\ 2+1+1+1,\ 1+1+1+1+1$ and those for $\mathcal {E}_{u}(5)$ are $5,\ 3+2,\ 3+1+1,\ 2+2+1,\ 2+1+1+1,\ 1+1+1+1+1.$

### Theorem 1.6

Let

$$\begin{aligned} \frac{\psi (q)}{\phi (q)} := \sum _{n=0}^{\infty }a_{\psi ,\phi }(n)q^n. \end{aligned}$$

Then, we have

1.8 $$\begin{aligned} p(n,4) = p_{ou,ed}(n) =\mathcal {E}_{\text {u}}(n) = (-1)^n a_{\psi ,\phi }(n). \end{aligned}$$

$\mathcal {O}_{d}(n)$ denote the number of partitions of *n* such that the odd parts are distinct and each positive odd integer smaller than the largest odd part must appear as a part of the partition. For example, the six partitions enumerated by $\mathcal {O}_{d}(9)$ are $8 + 1,\ 6 + 2 + 1,\ 4 + 4 + 1,\ 4 + 2 + 2 + 1,\ 5 + 3 + 1.$

### Theorem 1.7

$$\begin{aligned} \sum _{n=0}^{\infty }\mathcal {O}_{d}(2n+1)q^n = \frac{\psi (q^4)}{f(-q)}. \end{aligned}$$

### Theorem 1.8

$$\begin{aligned} c_{e,o}(n) \equiv p(n) \ \ (\text {mod} \ 2). \end{aligned}$$

Moreover based on the numerical evidences, it seems that for all $n \ge 0$,

$$\begin{aligned} c_{o}(2n) \equiv 0\ (\text {mod}\ 4). \end{aligned}$$

This has been checked up to $n = 2000$. We leave this as an open problem.

### Theorem 1.9

1.9 $$\begin{aligned} c_{e,o}(5n+4) \equiv 0 \ (\text {mod} \ 5) \end{aligned}$$

and

1.10 $$\begin{aligned} c_{o}(5n+4) \equiv 0 \ (\text {mod} \ 10). \end{aligned}$$

Following Fine’s notation [15, Ch. 2, Example 2], we define $p_{E}(n)$ ($p_{O}(n)$, respectively) to be the number of partitions of *n* into even (odd, respectively) number of parts. We recall one of the classical completely multiplicative function, Liouville’s function $\lambda$, defined by

$$\begin{aligned} \lambda (n)={\left\{ \begin{array}{ll} 1, &{}\quad \text {if}\, n=1, \\ (-1)^{a_{1}+\dots +a_{k}}, &{}\quad \text {if}\, n=p_{1}^{a_{1}}\dots p_{k}^{a_{k}}.\\ \end{array}\right. } \end{aligned}$$

### Theorem 1.10

For all $$n \in \mathbb {Z}_{\ge 2}$$,

$$\begin{aligned} c_{o}(n)=p_{O}(n)-(-1)^n \sum _{d|n}\lambda (d)+(-1)^n\sum _{k=0}^{n-2}\Bigl (\sum _{d|k+1}\lambda (d)\Bigr ) sc(n-k-1), \end{aligned}$$

where *sc*(*n*) denotes the number of self-conjugate partitions of *n*.

As our proof of Theorem 1.10 primarily relies on comparing coefficients of a certain *q*-series identity, we would like to ask if there a bijective proof of Theorem 1.10. Next we move on to the last theorem of this paper. Let $p (n, \Box )$ denote the number of partitions of *n*, where the side of the Durfee square does not occur as a part of the partition. For example, consider the partitions of 8: the partition 8 with side of Durfee square one and the partitions $5+3,\ 4+4,\ 4+3+1,\ 3+3+1+1$ with side of Durfee square two are altogether five partitions of 8 where the side of respective Durfee square is not a part of those partitions of 8.

Define $\mathfrak {F}_{0}(n)$ (resp. $\mathfrak {F}^{'}_{0}(n)$) to be the number of 0s in the Frobenius symbols in the partitions of *n* (resp. the numbers of Frobenius symbols for the partitions of *n* with no 0s). For instance, $\mathfrak {F}_{0}(8)=20$ and $\mathfrak {F}^{'}_{0}(7)=5$ enumerated by the Frobenius symbols

$$\begin{aligned} \Biggl \{\begin{pmatrix} 5 \\ 1 \end{pmatrix} , \begin{pmatrix} 4 \\ 2 \end{pmatrix} , \begin{pmatrix} 3 \\ 3 \end{pmatrix} , \begin{pmatrix} 2 \\ 4 \end{pmatrix} , \begin{pmatrix} 1 \\ 5 \end{pmatrix} \Biggr \}. \end{aligned}$$

### Theorem 1.11

$$p (n, \Box ) = \frac{1}{2}\mathfrak {F}_{0}(n) - \mathfrak {F}^{'}_{0}(n-1)$$.

## Proof of Theorems

### Proof of Theorem 1.6

We begin the proof with following identity

2.1 $$\begin{aligned} \sum _{n=0}^{\infty }p(n,4)q^n=\frac{(q^4,q^4)_{\infty }}{(q;q)_{\infty }} = \frac{(-q^2,q^2)_{\infty }}{(q;q^2)_{\infty }}= \sum _{n=0}^{\infty }p_{ou,ed}(n)q^n \end{aligned}$$

that establishes $p(n,4)=p_{ou,ed}(n)$. The generating function of $\mathcal {E}_{u}(n)$ is given by

2.2 $$\begin{aligned} \sum _{n=0}^{\infty }\mathcal {E}_{u}(n)q^n=\frac{1}{(q;q^2)_{\infty }}\sum _{n=0}^{\infty }\frac{q^{2+4+\dots +2n}}{(q^2;q^2)_{n}}=\frac{1}{(q;q^2)_{\infty }}\sum _{n=0}^{\infty }\frac{q^{n(n+1)}}{(q^2;q^2)_{n}}. \end{aligned}$$

We note that $\frac{1}{(q;q^2)_{\infty }}$ contributes to all the odd parts that occur in $\mathcal {E}_{u}(n)$ and $\frac{q^{2+4+\dots +2n}}{(q^2;q^2)_{n}}$ counts all those partitions in which even parts are unrestricted and every positive even integer smaller than the greatest even part occurs as a part. Applying $z\mapsto -q$ into the following identity [11, Corollary 1.3.2, Equation (1.3.7)]:

$$\begin{aligned} \sum _{n=0}^{\infty }\dfrac{(-z)^n q^{\frac{n(n-1)}{2}}}{(q;q)_n}=(z;q)_{\infty }, \end{aligned}$$

we obtain

2.3 $$\begin{aligned} \sum _{n=0}^{\infty }\frac{q^{\frac{n(n+1)}{2}}}{(q;q)_{n}} = (-q;q)_{\infty }. \end{aligned}$$

Now following the substitution $q\mapsto q^2$ in (2.3) and from (2.2), it follows that

2.4 $$\begin{aligned} \frac{1}{(q;q^2)_{\infty }}\sum _{n=0}^{\infty }\frac{q^{n(n+1)}}{(q^2;q^2)_{n}} = \dfrac{(-q^2;q^2)_{\infty }}{(q;q^2)_{\infty }}=\sum _{n=0}^{\infty }\mathcal {E}_{u}(n)q^n. \end{aligned}$$

So, (2.2) and (2.4) implies

2.5 $$\begin{aligned} \sum _{n=0}^{\infty }\mathcal {E}_{u}(n)q^n=\frac{(-q^2;q^2)_{\infty }}{(q;q^2)_{\infty }}. \end{aligned}$$

Putting down (2.1) and (2.5) together, it follows that

2.6 $$\begin{aligned} \sum _{n=0}^{\infty }p(n,4)q^n= \sum _{n=0}^{\infty }p_{ou,ed}(n)q^n=\sum _{n=0}^{\infty }\mathcal {E}_{u}(n)q^n=\frac{(q^4;q^4)_{\infty }}{(q;q)_{\infty }}. \end{aligned}$$

To prove the remaining part of (1.8), we start with

2.7 $$\begin{aligned} \sum _{n=0}^{\infty }a_{\psi ,\phi }(n)q^n=\frac{\psi (q)}{\phi (q)}=\frac{(q^4;q^4)^{2}_{\infty }}{(q^2;q^2)^{3}_{\infty }}(q;q)_{\infty }=\frac{(q^4;q^4)^{2}_{\infty }}{(q^2;q^2)^{3}_{\infty }} f(-q). \end{aligned}$$

Applying $q\mapsto -q$ into (2.7), we get

2.8 $$\begin{aligned} \sum _{n=0}^{\infty }(-1)^n a_{\psi ,\phi }(n)q^n=\frac{(q^4;q^4)^{2}_{\infty }}{(q^2;q^2)^{3}_{\infty }} f(q). \end{aligned}$$

Now

2.9 $$\begin{aligned} f(q)= & {} \dfrac{f(-q)}{\psi (-q)} \psi (q)\ \ \ \Bigl (\text {by\ \ [10, Entry 24 (i)]}\Bigr )\nonumber \\= & {} \dfrac{(q;q)_{\infty }(-q;q^2)_{\infty }}{(q^2;q^2)_{\infty }}\psi (q)= \dfrac{(q^2;q^2)_{\infty }}{(q^4;q^4)_{\infty }}\psi (q)\nonumber \\= & {} \dfrac{(q^2;q^2)_{\infty }}{(q^4;q^4)_{\infty }}\dfrac{(q^2;q^2)_{\infty }}{(q;q^2)_{\infty }}=\dfrac{(q^2;q^2)^3_{\infty }}{(q^4;q^4)_{\infty }(q;q)_{\infty }}. \end{aligned}$$

From (2.8) and (2.9), it follows that

2.10 $$\begin{aligned} \sum _{n=0}^{\infty }(-1)^n a_{\psi ,\phi }(n)q^n=\frac{(q^4;q^4)_{\infty }}{(q;q)_{\infty }}. \end{aligned}$$

The *q*-series identities (2.6) and (2.10) conclude the proof of Theorem 1.6.

### Proof of Theorem 1.7

From [3, Equation (3.1)], it follows that

2.11 $$\begin{aligned} \sum _{n=0}^{\infty }\mathcal {O}_{d}(n)q^n = \frac{1}{2(q^2;q^2)_{\infty }}\Bigl (1+\sum _{n=-\infty }^{\infty }q^{n^2}\Bigr )= & {} \frac{1}{2(q^2;q^2)_{\infty }}\Bigl (1+\phi (q)\Bigr )\nonumber \\= & {} \frac{1}{2(q^2;q^2)_{\infty }}\Bigl (1+f(q,q)\Bigr ). \nonumber \\ \end{aligned}$$

Applying (1.4) with $n=2$ and $a=b=q$, we have

2.12 $$\begin{aligned} f(q,q) = f(q^4,q^4)+q f(q^8,1). \end{aligned}$$

From (1.4) and (2.11), it follows that

2.13 $$\begin{aligned} \sum _{n=0}^{\infty }\mathcal {O}_{d}(n)q^n = \frac{1}{2(q^2;q^2)_{\infty }}+\frac{1}{2}\frac{f(q^4,q^4)}{(q^2;q^2)_{\infty }}+\frac{q}{2}\frac{f(q^8,1)}{(q^2;q^2)_{\infty }}, \end{aligned}$$

and therefore,

2.14 $$\begin{aligned} \sum _{n=0}^{\infty }\mathcal {O}_{d}(2n+1)q^{2n+1}= & {} \frac{q}{2}\frac{f(q^8,1)}{(q^2;q^2)_{\infty }} \nonumber \\= & {} \frac{q}{2 (q^2;q^2)_{\infty }}(-q^8;q^8)_{\infty }(-1;q^8)_{\infty }(q^8;q^8)_{\infty } \ \ (\text {by}\ \ (1.3) )\nonumber \\= & {} \frac{q}{ (q^2;q^2)_{\infty }}(-q^8;q^8)^{2}_{\infty }(q^8;q^8)_{\infty }. \end{aligned}$$

Dividing by *q* and then replacing $q^2$ by *q* in (2.14), we finally have

$$\begin{aligned} \sum _{n=0}^{\infty }\mathcal {O}_{d}(2n+1)q^{n}= \frac{(-q^4;q^4)^{2}_{\infty }(q^4;q^4)_{\infty }}{ (q;q)_{\infty }}= \frac{(q^8;q^8)_{\infty }}{(q^4;q^8)_{\infty }}\frac{1}{(q;q)_{\infty }}= \frac{\psi (q^4)}{f(-q)}, \end{aligned}$$

which finishes the proof of Theorem 1.7. $\square$

### Proof of Theorem 1.8

To prove Theorem 1.8, it suffices to show that

2.15 $$\begin{aligned} c_{o}(n) \equiv 0 \ (\text {mod} \ 2) \end{aligned}$$

as $c_{e}(n)+c_{o}(n)=p(n)$. Due to Euler [11, Equation (1.1.7)], we have

2.16 $$\begin{aligned} \sum _{n=0}^{\infty }p(n)q^n = \sum _{n=0}^{\infty }(c_{e}(n)+c_{o}(n))q^n= \frac{1}{(q;q)_{\infty }}. \end{aligned}$$

From [7, p. 168, Equation (1.11)] with $z=-1$, it follows that

2.17 $$\begin{aligned} \sum _{n=0}^{\infty }c_{e,o}(n)q^n := \sum _{n=0}^{\infty }(c_{e}(n)-c_{o}(n))q^n= \frac{(q;q)_{\infty }}{(-q;q)^{2}_{\infty }} = \phi (-q)\chi (-q). \end{aligned}$$

By (2.17) and (2.16), we have

2.18 $$\begin{aligned} \sum _{n=0}^{\infty }c_{o}(n)q^n=\frac{1}{2}\Bigl (\frac{1}{(q;q)_{\infty }}-\frac{(q;q)_{\infty }}{(-q;q)^{2}_{\infty }}\Bigr ). \end{aligned}$$

From [5, Entry 3.1.1] with $a=-1$, it follows that

2.19 $$\begin{aligned} \frac{(q;q)_{\infty }}{(-q;q)^{2}_{\infty }} =\frac{1}{(q;q)_{\infty }} \Biggl (1- \underset{n=0}{\sum _{m=1}^{\infty }}(-1)^m q^{\frac{m(m+1)}{2}+mn}\Bigl (A_{n+1}-A_{n}\Bigr ) \Biggr ) \end{aligned}$$

with

2.20 $$\begin{aligned} A_{n+1}-A_{n} = 4(-1)^{n+1}. \end{aligned}$$

Substituting (2.19) and (2.20) into (2.18), we have

2.21 $$\begin{aligned} \sum _{n=0}^{\infty }c_{o}(n)q^n&= \frac{2}{(q;q)_{\infty }}\underset{n=0}{\sum _{m=1}^{\infty }}(-1)^{m+n+1} q^{\frac{m(m+1)}{2}+mn} \nonumber \\&= \frac{2}{(q;q)_{\infty }}\sum _{m=1}^{\infty }(-1)^{m+1}\frac{ q^{\frac{m(m+1)}{2}}}{1+q^m}. \end{aligned}$$

Now, it can be easily observed that the right hand side of the above equation is of the form $\sum _{n=0}^{\infty }a_n q^n$ with $(a_n)_{n \ge 0}$ a sequence of even integers; i.e., $c_o(n) \equiv 0\ (\text {mod}\ 2)$ which concludes the proof of Theorem 1.8. $\square$

### Proof of Theorem 1.9

We shall make use of the 5-dissections of $(q;q)^{3}_{\infty }$ and $\frac{1}{(q^2;q^2)^2_{\infty }}$ as we rewrite (2.17) in the following way:

2.22 $$\begin{aligned} \sum _{n=0}^{\infty }c_{e,o}(n)q^n= \frac{(q;q)^3_{\infty }}{(q^2;q^2)^{2}_{\infty }}. \end{aligned}$$

Applying (1.6) of Lemma 1.2, we have

2.23 $$\begin{aligned} (q;q)^{3}_{\infty } = A_{0}+A_{1}+A_{3}, \end{aligned}$$

where $A_{i}$ consists of terms in which powers of *q* congruent to *i* modulo 5 can be written as follows

2.24 $$\begin{aligned} A_{0}= & {} (q^{25};q^{25})^{3}_{\infty }\Bigl (T^{3}(q^5)-\frac{3q^5}{T^{2}(q^5)}\Bigr ),\nonumber \\ A_{1}= & {} -q (q^{25};q^{25})^{3}_{\infty }\Bigl (3 T^{2}(q^5)+\frac{q^5}{T^{3}(q^5)}\Bigr ),\nonumber \\ A_{3}= & {} 5q^3 (q^{25};q^{25})^{3}_{\infty }. \end{aligned}$$

For the 5-dissection of $\frac{1}{(q^2;q^2)^2_{\infty }}$, first let $q \mapsto q^2$ and then by squaring both sides of (1.7), it follows that

2.25 $$\begin{aligned} \begin{aligned} \frac{1}{(q^2;q^2)^2_{\infty }}=&\, \frac{(q^{50};q^{50})^{10}_{\infty }}{(q^{10};q^{10})^{12}_{\infty }}\Biggl (T^{8}(q^{10})+2q^2 \ T^{7}(q^{10})+5q^4 \ T^{6}(q^{10})+10q^6 \ T^{5}(q^{10})\\&+20q^8 \ T^{4}(q^{10})+16q^{10} \ T^{3}(q^{10})+27q^{12} \ T^{2}(q^{10})+20q^{14} \ T(q^{10})\\&+15q^{16}-20\frac{q^{18}}{T(q^{10})}+27\frac{q^{20}}{T^{2}(q^{10})}-16\frac{q^{22}}{T^{3}(q^{10})}+20\frac{q^{24}}{T^{4}(q^{10})}\\&-10\frac{q^{26}}{T^{5}(q^{10})}+5\frac{q^{28}}{T^{6}(q^{10})}-2\frac{q^{30}}{T^{7}(q^{10})}+\frac{q^{32}}{T^{8}(q^{10})}\Biggr ).\\ \end{aligned} \end{aligned}$$

Similar to (2.23), we write

2.26 $$\begin{aligned} \frac{1}{(q^2;q^2)^2_{\infty }} := B_{0}+B_{1}+B_{2}+B_{3}+B_{4}, \end{aligned}$$

with

2.27 $$\begin{aligned} B_{1}= & {} 5 q^{6}\frac{(q^{50};q^{50})^{10}_{\infty }}{(q^{10};q^{10})^{12}_{\infty }}\Bigl (2 \ T^{5}(q^{10})+3q^{10}-2 \ \frac{q^{20}}{T^{5}(q^{10})}\Bigr ),\nonumber \\ B_{3}= & {} 5 q^{8}\frac{(q^{50};q^{50})^{10}_{\infty }}{(q^{10};q^{10})^{12}_{\infty }}\Bigl (2 \ T^{2}(q^{10})- \frac{q^{10}}{T^{3}(q^{10})}\Bigr )^{2},\nonumber \\ B_{4}= & {} 5 q^{4}\frac{(q^{50};q^{50})^{10}_{\infty }}{(q^{10};q^{10})^{12}_{\infty }}\Bigl ( T^{3}(q^{10})+ \frac{q^{10}}{T^{2}(q^{10})}\Bigr )^{2}.\nonumber \\ \end{aligned}$$

Consequently by (2.23) and (2.26), it follows that

2.28 $$\begin{aligned} \sum _{n=0}^{\infty }c_{e,o}(5n+4)q^{5n+4}=A_{0}B_{4}+A_{1}B_{3}+A_{3}B_{1}. \end{aligned}$$

Finally, we plug in (2.24) and (2.27) into (2.28), and then substitute $q^{5} \mapsto q$. This is followed by division on both side by $q^{4}$, and hence we obtain:

2.29 $$\begin{aligned}{} & {} \sum _{n=0}^{\infty }c_{e,o}(5n+4)q^{n}= 5 \frac{(q^{5};q^{5})^{3}_{\infty }(q^{10};q^{10})^{10}_{\infty }}{(q^{2};q^{2})^{12}_{\infty }}\Biggl (\Bigl (T^{3}(q)-3 \frac{q}{T^{2}(q)}\Bigr )\nonumber \\{} & {} \quad \Bigl (T^{3}(q^2)+\frac{q^2}{T^{2}(q^2)}\Bigr )^{2}-q \Bigl (3 T^{2}(q)+ \frac{q}{T^{3}(q)}\Bigr )\Bigl (2T^{2}(q^2)-\frac{q^2}{T^{3}(q^2)}\Bigr )^{2}\nonumber \\{} & {} \quad +5 q\Bigg (2T^{5}(q^2)+3q^2-2\frac{q^4}{T^{5}(q^2)}\Bigg ) \Biggr ). \end{aligned}$$

This implies that $c_{e,o}(5n+4) \equiv 0\ (\text {mod}\ 5)$ as claimed in (1.9). Now,

$$\begin{aligned} c_{e,o}(5n+4)=c_{e}(5n+4)-c_{o}(5n+4) \equiv 0\ (\text {mod}\ 5) \end{aligned}$$

and

$$\begin{aligned} p(5n+4)=c_{e}(5n+4)+c_{o}(5n+4) \equiv 0\ (\text {mod}\ 5)\ \ (\text {by}\ \ (2.16)\ \text {and}\ (1.1)) \end{aligned}$$

imply that for all $n \ge 0$,

2.30 $$\begin{aligned} c_{o}(5n+4) \equiv 0\ (\text {mod}\ 5). \end{aligned}$$

We have already proved that for all $n \ge 0$, $c_{o}(n) \equiv 0\ (\text {mod}\ 2)$, see (2.15), which in particular states that for all $n \ge 0$,

2.31 $$\begin{aligned} c_{o}(5n+4) \equiv 0\ (\text {mod}\ 2). \end{aligned}$$

From (2.30) and (2.31), it follows that $c_{o}(5n+4) \equiv 0\ (\text {mod}\ 10)$ which finishes the proof of (1.10). $\square$

### Proof of Theorem 1.10

From [15, Ch. 2, Equation (22.14)], we get

2.32 $$\begin{aligned} \sum _{n=0}^{\infty } (p_{E}(n)-p_{O}(n))q^n =\frac{1}{(-q;q)_{\infty }}. \end{aligned}$$

As $p_{E}(n)+p_{O}(n)=p(n)$ and $\sum _{n=0}^{\infty }p(n)q^n=1/(q;q)_{\infty }$, from (2.32), it follows that

2.33 $$\begin{aligned} \sum _{n=0}^{\infty } p_{O}(n)q^n =\dfrac{1}{2}\Biggl (\frac{1}{(q;q)_{\infty }}-\frac{1}{(-q;q)_{\infty }}\Biggr ). \end{aligned}$$

Due to Glaisher [17, XVI, p. 256],

2.34 $$\begin{aligned} \sum _{n=0}^{\infty } (p_{E}(n)-p_{O}(n))q^n =\sum _{n=0}^{\infty }(-1)^n sc(n)q^n=\frac{1}{(-q;q)_{\infty }}. \end{aligned}$$

Recall the identity due to Gauss [1, Corollary 2.10, Equation (2.2.12)] which states that

2.35 $$\begin{aligned} \frac{(q;q)_{\infty }}{(-q;q)_{\infty }} = \sum _{n=-\infty }^{\infty }(-1)^n q^{n^2}=1+2\sum _{n=1}^{\infty }(-1)^nq^{n^2}. \end{aligned}$$

Now

2.36 $$\begin{aligned} \sum _{n=0}^{\infty }c_{o}(n)q^n= & {} \frac{1}{2}\Bigl (\frac{1}{(q;q)_{\infty }}-\frac{(q;q)_{\infty }}{(-q;q)^{2}_{\infty }}\Bigr )\ \ (\text {by}\ \ (2.18))\nonumber \\= & {} \frac{1}{2}\Bigl (\frac{1}{(q;q)_{\infty }}-\frac{(q;q)_{\infty }}{(-q;q)_{\infty }}\frac{1}{(-q;q)_{\infty }}\Bigr ) \nonumber \\= & {} \frac{1}{2}\Biggl (\frac{1}{(q;q)_{\infty }}-\Bigl (1+2\sum _{n=1}^{\infty }(-1)^nq^{n^2}\Bigr )\frac{1}{(-q;q)_{\infty }}\Biggr ) \ (\text {by}\ \ (2.35)) \nonumber \\= & {} \frac{1}{2}\Biggl (\frac{1}{(q;q)_{\infty }}-\frac{1}{(-q;q)_{\infty }}\Biggr )-\dfrac{1}{(-q;q)_{\infty }}\sum _{n=1}^{\infty }(-1)^nq^{n^2}\nonumber \\= & {} \sum _{n=0}^{\infty }p_{O}(n)q^n-\dfrac{1}{(-q;q)_{\infty }}\sum _{n=1}^{\infty }(-1)^nq^{n^2}\ \ (\text {by}\ \ (2.33))\nonumber \\= & {} \sum _{n=0}^{\infty }p_{O}(n)q^n-\sum _{n=1}^{\infty }(-1)^nq^{n^2}\sum _{n=0}^{\infty }(-1)^n sc(n)q^n\ \ (\text {by}\ \ (2.34))\nonumber \\= & {} \sum _{n=0}^{\infty }p_{O}(n)q^n-\sum _{n=1}^{\infty }(-1)^nq^{n^2}\Biggl (1+\sum _{n=1}^{\infty }(-1)^n sc(n)q^n\Biggr )\nonumber \\= & {} \sum _{n=0}^{\infty }p_{O}(n)q^n-\sum _{n=1}^{\infty }(-1)^nq^{n^2}-\sum _{n=1}^{\infty }(-1)^nq^{n^2}\sum _{n=1}^{\infty }(-1)^n sc(n)q^n. \nonumber \\ \end{aligned}$$

From (2.36), for all $n \in \mathbb {Z}_{\ge 2}$, it follows that

2.37 $$\begin{aligned} c_{o}(n)= p_{O}(n)-(-1)^n\delta _{(n,\Box )}+ (-1)^n\sum _{k=0}^{n-2}\delta _{(k+1,\Box )} \ sc(n-k-1), \end{aligned}$$

where

$$\begin{aligned} \delta _{(m,\Box )}= {\left\{ \begin{array}{ll} 1, &{}\quad \text {if}\, m \, \text {is a square}, \\ 0, &{}\quad \text {otherwise}.\\ \end{array}\right. } \end{aligned}$$

Due to [4, Theorem 2.19], we know that

2.38 $$\begin{aligned} \delta _{(m,\Box )}=\sum _{d|m}\lambda (d). \end{aligned}$$

Combining (2.37) and (2.38), we conclude the proof of Theorem 1.10. $\square$

### Proof of Theorem 1.11

Due to Euler [1, Corollary 2.6, Equation (2.2.9)], we have

2.39 $$\begin{aligned} \frac{1}{(q;q)_{\infty }}=\sum _{n=0}^{\infty }\frac{q^{n^2}}{(q;q)^{2}_{n}}, \end{aligned}$$

where the $n^{\text {th}}$ term of (2.39) $\frac{q^{n^2}}{(q;q)^{2}_{n}}$ is the generating function for those partitions with Durfee square of side *n*. We observe that the generating function for $p (n, \Box )$ is

2.40 $$\begin{aligned} \sum _{n=0}^{\infty }p (n, \Box )q^n= & {} \sum _{n=0}^{\infty }\frac{q^n\cdot q^{n^2}}{(1-q^n)(q;q)^{2}_{n-1}} \nonumber \\= & {} \sum _{n=0}^{\infty }\frac{q^{n^2+n}(1-q^n)}{(q;q)^{2}_{n}}\nonumber \\= & {} \sum _{n=0}^{\infty }\frac{q^{n^2+n}}{(q;q)^2_{n}}-\sum _{n=0}^{\infty }\frac{q^{n^2+2n}}{(q;q)^2_{n}}. \end{aligned}$$

For $j \in \mathbb {Z}$, associated with enumeration of crank statistics given in (1.4), we define

$$\begin{aligned} \begin{aligned} M_{\ge j}(n)&:= |\{\pi \vdash n: c(\pi ) \ge j \}| \ \ \text {and} \ \ M_{j}(n) := |\{\pi \vdash n: c(\pi )= j \}|. \end{aligned} \end{aligned}$$

By [18, Proposition 6 and Theorem 7], we rewrite the last line of (2.40) as

2.41 $$\begin{aligned} \sum _{n=0}^{\infty }p (n, \Box )q^n = \sum _{n=0}^{\infty }M_{\ge 0}(n)q^n-\sum _{n=0}^{\infty }\mathfrak {F}^{'}_{0}(n)q^n. \end{aligned}$$

Finally, we conclude the proof of Theorem 1.11 by showing that

$$\begin{aligned} p (n, \Box ) = M_{\ge 0}(n)-\mathfrak {F}^{'}_{0}(n)&= M_{ 0}(n)+M_{\ge 1}(n)-\mathfrak {F}^{'}_{0}(n)\\&= M_{\ge 1}(n)-\mathfrak {F}^{'}_{0}(n-1) \ \ \Bigl (\text {by\ \ [18, Proposition 6]}\Bigr )\\&= \frac{1}{2}\mathfrak {F}_{0}(n) - \mathfrak {F}^{'}_{0}(n-1) \ \ \Bigl (\text {by\ \ [6, Theorem 2]}\Bigr ).\\ \end{aligned}$$

$$\square $$

## Data Availability

No data were generated or used in the preparation of this paper.
